# Negative regulation of ATP-induced inflammasome activation and cytokine secretion by acute-phase proteins: A mini review

**DOI:** 10.3389/fphar.2022.981276

**Published:** 2022-08-29

**Authors:** Katrin Richter, Anca-Laura Amati, Winfried Padberg, Veronika Grau

**Affiliations:** Laboratory of Experimental Surgery, Department of General and Thoracic Surgery, German Center for Lung Research, Justus-Liebig-University, Giessen, Germany

**Keywords:** α1-antitrypsin, ATP, CHRNA7, CHRNA9, CHRNA10, C-reactive protein, NLRP3 inflammasome, secretory leukocyte protease inhibitor

## Abstract

The expression of the acute-phase reactants C-reactive protein (CRP), α1-antitrypsin (AAT), and secretory leukocyte protease inhibitor (SLPI), is induced in response to inflammation by pro-inflammatory mediators, including interleukin-1β. It is conceivable that acute-phase proteins exert protective functions, when the integrity of an organism is challenged by pathogens or trauma, which result in uncontrolled release of endogenous damage-associated molecular patterns like Toll-like receptor agonists and ATP. Acute-phase proteins can enhance or down-modulate immunity against infections or protect the host against damage caused by over-shooting effector functions of the immune system. CRP is mainly regarded as a pro-inflammatory opsonizing agent that binds to bacteria and damaged host cells thereby contributing to their inactivation and elimination. AAT and SLPI are well known for their anti-protease activity, which protects the lung extracellular matrix against degradation by proteases that are released by activated neutrophil granulocytes. In addition, there is growing evidence, that CRP, AAT, and SLPI can control the biosynthesis, maturation, and secretion of pro-inflammatory cytokines. The purpose of this narrative mini review is to summarize these anti-inflammatory functions with a focus on the negative control of the ATP-induced, inflammasome-dependent secretion of interleukin-1β by monocytes. CRP-, AAT- and SLPI-mediated control of interleukin-1β release involves the activation of unconventional nicotinic acetylcholine receptors that inhibits the ionotropic function of the ATP receptor P2X7. Apart from other functions, CRP, AAT, and SLPI seem to be central elements of systemic negative feedback loops that protect the host against systemic hyperinflammation, barrier dysfunction, and death by multiple organ damage.

## Introduction

During systemic inflammation caused by trauma, infection, and autoinflammatory or autoimmune diseases, circulating pro-inflammatory mediators trigger acute-phase responses including the synthesis and release of the acute-phase reactants C-reactive protein (CRP), α1-antitrypsin (AAT), and secretory leukocyte protease inhibitor (SLPI) (Gabay and Kushner, 1999; Douglas and Hannila, 2022). These acute-phase proteins exert pleiotropic functions including the facilitation of pathogen clearance, protection of the host from the attack of proteases mainly released from activated neutrophil granulocytes, and the modulation of inflammation. In this narrative mini review, we will give a brief overview of the anti-inflammatory effects of CRP, AAT, and SLPI with a focus on our own data regarding the control of ATP-induced inflammasome assembly, maturation, and release of interleukin (IL)-1β, which is mediated by nicotinic acetylcholine receptors (nAChRs). A literature search *via* PubMed® including the terms “ATP,” “inflammasome,” “nicotinic,” and “control” retrieved 10 hits. Among them, only three research papers dealt with acute-phase proteins, which are discussed here in more detail (Richter et al., 2018; Siebers et al., 2018; Zakrzewicz et al., 2019).

IL-1β and IL-6, an IL-1β-inducible cytokine, are among the most important inducers of the acute-phase response (Ramadori and Christ, 1999). IL-1β is a potent pro-inflammatory, pyrogenic cytokine expressed by numerous cell types including monocytes, macrophages, and neutrophil granulocytes (Mantovani et al., 2019), that importantly contributes to host defense against pathogens. However, excessive secretion of IL-1β can cause severe inflammatory and autoimmune disorders (Mantovani et al., 2019). IL-1β released in response to extended traumatic cell damage has the potential to induce severe systemic inflammation culminating in barrier dysfunction, sepsis, and in a life-threatening multi-organ dysfunction syndrome (Mantovani et al., 2019). As during evolution, trauma and infection presumably were among the leading causes of death, it is conceivable that complex and redundant mechanisms evolved that tightly regulate the biosynthesis, maturation, and secretion of IL-1β.

Like other pro-inflammatory cytokines, the biosynthesis of pro-IL-1β is typically induced by the activation of pattern recognition receptors such as Toll-like receptors (TLRs) by danger-associated or pathogen-associated molecular patterns (DAMPs or PAMPs) that signal via the transcription factors nuclear factor-κB (NF-κB) or activator protein 1 (AP-1) (Mantovani et al., 2019). While several other cytokines are released upon biosynthesis, pro-IL-1β is not bioactive, devoid of a signal peptide and stays within the cytoplasm unless a second danger signal induces the assembly of inflammasomes. Inflammasomes are a family of multi-protein complexes that catalyze the activation of caspases, which finally cleave pro-IL-1β and enable the swift release of mature, bioactive IL-1β (Mantovani et al., 2019; Swanson et al., 2019). In the context of trauma, extracellular ATP typically activates the ATP-sensitive P2X7 receptor (P2X7R), a ligand-gated ion channel that induces the assembly of the NLRP3 (NACHT, LRR and PYD domains-containing protein 3) inflammasome, activation of caspase-1, and the secretion of mature IL-1β. IL-18 is another inflammasome-dependent pro-inflammatory cytokine often released by monocytes and macrophages along with IL-1β (Swanson et al., 2019).

Previously, we demonstrated that the ATP-induced ionotropic function of the P2X7R and, hence, IL-1β release by human monocytes can be inhibited by activation of nAChRs composed of subunits α7, α9, and α10 (Hecker et al., 2015). Apart from classical nAChR agonists including acetylcholine or nicotine, phosphocholine and diverse compounds containing a phosphocholine group namely glycerophosphocholine, lysophosphatidylcholine, and diplamitoyl-phosphatidylcholine can function as unconventional agonists of monocytic nAChRs (Hecker et al., 2015; Richter et al., 2016; Backhaus et al., 2017; Zakrzewicz et al., 2017). These unconventional nAChR agonists induce a metabotropic signaling to inhibit the ionotropic P2X7R function in monocytes but do not trigger the well-known ligand-gated ion currents of nAChRs (Richter et al., 2016; Zakrzewicz et al., 2017). Of note, ATP-independent mechanisms of inflammasome activation are not affected by nAChR (Hecker et al., 2015). Interestingly, the anti-inflammatory effects of nAChR activation are countered by high concentrations of amyloid-β (Richter et al., 2020). Recent findings provide evidence that the acute-phase reactants CRP (Richter et al., 2018), AAT (Siebers et al., 2018), and SLPI (Zakrzewicz et al., 2019) potently activate nAChRs at human monocytic cells and efficiently inhibit the ATP-induced, inflammasome-dependent IL-1β release, suggesting that these acute-phase proteins are a part of negative feed-back loops controlling acute inflammation.

### C-reactive protein

CRP belongs to the family of pentraxins and is a sensitive clinical marker for inflammation (Pepys and Hirschfield, 2003; Mantovani et al., 2008). In response to circulating IL-1β and IL-6 the biosynthesis and secretion of CRP is strongly induced in hepatocytes (Pepys and Hirschfield, 2003; Mantovani et al., 2008). Systemic CRP levels can raise up to 1,000-fold within two to 3 days and can reach concentrations above 300 μg/ml blood (Pepys and Hirschfield, 2003; Mantovani et al., 2008). During evolution, the CRP gene was highly conserved, and no CRP-deficient individuals have ever been reported, suggesting a life-supporting biological function (Pepys and Hirschfield, 2003). Native CRP, which is circulating in the blood stream, consists of homomeric subunits that assemble to a doughnut-shaped pentamer. On one side of the pentamer, each subunit contains a Ca^2+^-dependent binding site for phosphocholine or other molecules containing a phosphocholine head-group including phosphatidylcholines and lysophosphatidylcholines (Pepys and Hirschfield, 2003). Although some reports implicate that native CRP can bind small soluble molecules with a choline head-group (Xia and Samols, 1997; Black et al., 2005; Nazarov et al., 2007; Richter et al., 2018), it is unclear, if native circulating CRP is ligand-laden. Binding of CRP to surfaces exposing phosphocholine, such as damaged cells or some pathogens, activates CRP, which results in conformational changes and monomerization (Sproston and Ashworth, 2018; Zeller et al., 2022). Upon activation, CRP exposes binding sites for Fc receptors and complement, which mediate effector functions including an increased mRNA and protein expression of pro-inflammatory cytokines and components of the NLRP3 inflammasome (Sproston and Ashworth, 2018; Zeller et al., 2022). In addition, CRP might induce NLRP3 inflammasome assembly *via* Fcγ receptor-mediated production of reactive oxygen species (Bian et al., 2019) and at least theoretically via complement activation and formation of the membrane attack complex (Xie et al., 2020). Hence, CRP locally enhances host defense against pathogens and facilitates clearing of apoptotic or otherwise damaged cells. Activated CRP can, however, also induce cell and tissue damage by activating the complement system, which plays a pathogenic role in acute myocardial infarction (Sproston and Ashworth, 2018; Bian et al., 2019; Zeller et al., 2022).

Anti-inflammatory functions of CRP, which seem to be mainly mediated by the native pentameric form of CRP (Sproston and Ashworth, 2018), are less well understood, and presumably mediated by diverse mechanisms. Native CRP can counter complement activation induced by monomeric CRP (Sproston and Ashworth, 2018) and protect from lipopolysaccharide (LPS)-induced mortality by neutralizing platelet activating factor (PAF), most probably by interaction of PAF with the phosphocholine binding site of CRP (Xia and Samols, 1997; Black et al., 2005). CRP can induce the expression and secretion of the anti-inflammatory IL-1 receptor antagonist in human blood cells (Tilg et al., 1993), that potently antagonizes effects mediated by IL-1. In addition to a direct suppression of Th1 cell differentiation, binding of CRP to the inhibitory FCγ receptor IIB inhibits the maturation of dendritic cells and may contribute to the maintenance of peripheral T cell tolerance during systemic inflammation (Jimenez et al., 2018). Interestingly, transgenic animals overexpressing human CRP are protected from diverse inflammatory diseases (Mold et al., 2002; Jiang et al., 2006; Torzewski et al., 2014). These data were questioned because pro-inflammatory functions of human CRP might not be activated due to interspecies incompatibilities. However, these studies support the notion that human CRP has a strong anti-inflammatory potential.

Our laboratory demonstrated recently that native CRP isolated from human bodily fluids dose-dependently inhibits the ATP-induced NLRP3 inflammasome assembly and release of monocytic IL-1β by activation of nAChRs containing subunits α7, α9, and α10 (Richter et al., 2018). The IC_50_ of CRP (4.9 μg/ml) corresponds to marginally elevated blood CRP levels, which are typical for mild inflammation (Richter et al., 2018). The dose-effect curve of CRP is very steep and 10 μg/ml, which is a typical concentration in patients with a localized, contained inflammation, are sufficient to fully inhibit the ATP-induced release of IL-1β *in vitro* (Richter et al., 2018) (Table 1). The activity of CRP relies on its Ca^2+^-dependent interaction with a not yet identified endogenous soluble ligand, presumably phosphocholine or a compound with a phosphocholine head-group (Richter et al., 2018). CRP that has been depleted of its ligand by a Ca^2+^ chelator is inactive, but its activity can be reconstituted with Ca^2+^ and phosphocholine at low concentrations, which are ineffective in the absence of CRP (Richter et al., 2018). Thus, CRP and phosphocholine act synergistically on monocytic nAChRs. As already mentioned for other nAChR agonists, the negative effect of CRP on ATP-mediated IL-1β is countered by amyloid-β (Richter et al., 2020). In a small prospective cohort of patients with multiple traumata, high CRP levels at admission to the hospital correlated with low circulating IL-1β levels on the next day (Richter et al., 2018). A similar negative correlation of high pre-operative CRP levels and post-operative fever was seen in a retrospective cohort of women undergoing lung cancer surgery (Meyer et al., 2020). Altogether, these data suggest, that ligand-laden native CRP can function as a negative feed-back regulator of the ATP-dependent production of mature IL-1β by human monocytes.

**TABLE 1:** Requirements and composition of nAChR subunits necessary for nicotinic signaling of acute-phase proteins in monocytic cells.

Acute-phase protein	Required nAChR subunits	Reference
CRP	α7, α9, α10	Richter et al. (2018)
AAT	α9 and α7 or α10	Siebers et al. (2018)
SLPI	α7, α9, α10	Zakrzewicz et al. (2019)

AAT, α1-antitrypsin; CRP, native pentameric C-reactive protein; nAChRs, nicotinic acetylcholine receptors SLPI, secretory leukocyte protease inhibitor.

### Alpha1-antitrypsin

AAT is the prototypical serine protease inhibitor, also called SERPINA1. Hepatocytes constitutively produce AAT, which is secreted to the blood stream and reaches concentrations in the range of 1–2 mg/ml (Janciauskiene et al., 2011). In addition, monocytes, macrophages, neutrophil granulocytes, and diverse epithelial cells are minor sources of AAT that can increase local AAT concentrations in inflamed tissues. AAT is classified as an acute-phase proteins, since circulating pro-inflammatory mediators enhance AAT expression, resulting in an about fourfold increase in plasma levels (Janciauskiene et al., 2011).

Numerous proteases including neutrophil elastase, proteinase-3, trypsin, kallikrein-7, kallikrein-14, and matriptases are functionally inhibited by AAT (Ehlers, 2014). In addition to its anti-protease function, AAT is a versatile modulator of immune functions. Pro-inflammatory functions are mainly mediated by oxidized AAT and multimeric structures formed by mutated AAT, while AAT exerts pronounced anti-inflammatory functions in mononuclear phagocytes and neutrophil granulocytes. AAT scavenges oxygen radicals, protects from experimental ischemia-reperfusion injury, allograft rejection, and rheumatoid arthritis (Blanco et al., 2011; Ehlers, 2014; Edinger et al., 2021; Nakagiri et al., 2021). AAT up-regulates the expression of the anti-inflammatory mediators IL-10 and IL-1 receptor antagonist, while modulating the expression of TLRs and pro-inflammatory cytokines including IL-1β (Janciauskiene et al., 2011; Ehlers, 2014; McElvaney et al., 2020; Schuster et al., 2020). When applied together with pro-inflammatory stimuli such as LPS, AAT does not prevent but rather accelerates the expression of pro- and anti-inflammatory mediators, resulting in an early induction of an anti-inflammatory milieu and in an early down-regulation of the expression pro-inflammatory cytokines (Janciauskiene et al., 2007; Schuster et al., 2020). The exact molecular mechanisms, by which AAT modulates the innate immune system, are not yet fully understood. At least some of the mechanisms rely on complex interactions with numerous binding partners and some of the anti-inflammatory functions of AAT seem to be independent of its anti-protease function and can even be mediated by complexes formed by proteases and AAT (Potere et al., 2019; O'Brien et al., 2022). Anti-inflammatory effects of AAT are in part mediated by the lipoprotein receptor-related protein 1 (LRP1), which is activated by a sequence of amino acids of AAT that is exposed upon interaction of AAT with serine proteases (Potere et al., 2019).

Like CRP, AAT isolated from the blood of healthy human volunteers dose-dependently inhibits the ATP-induced IL-1β release by human monocytic cells with an IC_50_ of about 0.1 mg/ml (Siebers et al., 2018). Although AAT is readily taken up by cells and might end up in the cytoplasm (Janciauskiene et al., 2011), AAT does not interfere with the proteolytic activity of caspase-1 or other proteases to inhibit the maturation of IL-1β (Rahman et al., 2015). Instead, we observed that the lipid scavenger receptor CD36 and calcium-independent phospholipase A2β (iPLA2β) are involved in signaling. Activation of iPLA2β results in the secretion of yet unknown nAChR agonists. Finally, activation of nAChR inhibits the ATP-induced release of monocytic IL-1β (Siebers et al., 2018). However, in contrast to CRP, only the nAChR subunit α9 is essential for signaling, while subunits α7 and α10 can functionally replace each other (Siebers et al., 2018) (Table 1). This anti-inflammatory function of AAT is not only independent of its anti-protease function but also independent of its anti-oxidant function, because fully oxidized AAT efficiently inhibits the release of IL-1β (Siebers et al., 2018). By contrast, chemically reduced AAT, which is found in the AAT preparations Zemaira® and Respreeza®, is inactive but can be re-activated by oxidation (Siebers et al., 2018; Agné et al., 2021).

### Secretory leukocyte protease inhibitor

SLPI is a small 11.7 kDa monomeric protein that is constitutively produced and secreted by numerous epithelial cells, especially those lining the inner surfaces of the body, as well as by hepatocytes, neutrophil granulocytes, monocytes, and macrophages (Moreau et al., 2008). LPS, IL-1β, TNF-α, and thymic stromal lymphopoietin can further enhance the SLPI expression and increase its concentration in various bodily fluids, which qualifies SLPI as an acute-phase protein (Sallenave et al., 1994; Jin et al., 1997). While circulating SLPI levels under steady-state conditions are as low as 40 ng/ml, about 10 μg/ml are measured in the pulmonary epithelial lining fluid or in the saliva (Grobmyer et al., 2000; Weldon et al., 2007). Much like AAT, SLPI inhibits serine proteases including neutrophil elastase, cathepsin G, and trypsin and protects pulmonary elastic fibers from degradation (Williams et al., 2006). Independent of its anti-protease function, SLPI has protective anti-microbial, anti-inflammatory, and tolerogenic properties, while pro-inflammatory functions have not been described (Nugteren and Samsom, 2021; Douglas and Hannila, 2022). SLPI is involved in wound healing, it regulates the formation of neutrophil extracellular traps, inhibits apoptosis, and regulates cell proliferation (Nugteren and Samsom, 2021; Douglas and Hannila, 2022). Accordingly, SLPI exerts modulatory and protective functions in numerous human and experimental diseases, including endotoxemia, infection, psoriasis, asthma, neuro-degeneration, obesity, diabetes type 2, and cancer (Taggart et al., 2002; Taggart et al., 2005; Douglas and Hannila, 2022).

Mechanistically, SLPI attenuates the activation of monocytes and macrophages by PAMPs and DAMPs in several ways. It physically interacts with CD14, which is together with TLR4 an essential part of the LPS receptor complex, and thereby interferes with LPS sensing (Ding et al., 1999). Like AAT, SLPI is rapidly taken up by monocytes, where it can inhibit the proteolytic degradation of the inhibitor of κB and, hence, prevent the activation of NF-κB and the synthesis of pro-inflammatory cytokines (Taggart et al., 2002; Taggart et al., 2005; Douglas and Hannila, 2022). In addition, SLPI can localize to cell nuclei and repress the function of pro-inflammatory genes by competing for NF-κB p 65 binding sites (Taggart et al., 2005; Douglas and Hannila, 2022).

Our laboratory demonstrated that, much like AAT, physiologic concentrations of SLPI (IC_50_ about 0.1 μg/ml) induce an iPLA2β-dependent release of small bioactive factors that activate monocytic nAChRs and inhibit the ATP-induced NLRP3 inflammasome assembly and release of IL-1β (Zakrzewicz et al., 2019). In this context, cell surface-bound annexin A2 seems to function as a receptor for SLPI (Zakrzewicz et al., 2019). The bioactive factors that are released by monocytic cells in response to SLPI require nAChR subunits α7, 9, and 10 for signaling, which is in contrast to AAT, for which only nAChR subunit α9 is essential (Zakrzewicz et al., 2019) (Table 1). We assume that the nAChR agonists released in response to SLPI and AAT are similar but different, because we have shown before, that compounds with a phosphocholine head-group differ in their nAChR subunit requirements depending on the residues covalently linked to phosphocholine (Zakrzewicz et al., 2017). Due to its higher local concentrations, SLPI is expected to be exclusively active at mucosal surfaces, whereas CRP and AAT are supposed to be also active in the blood.

## Conclusion

**FIGURE 1:**
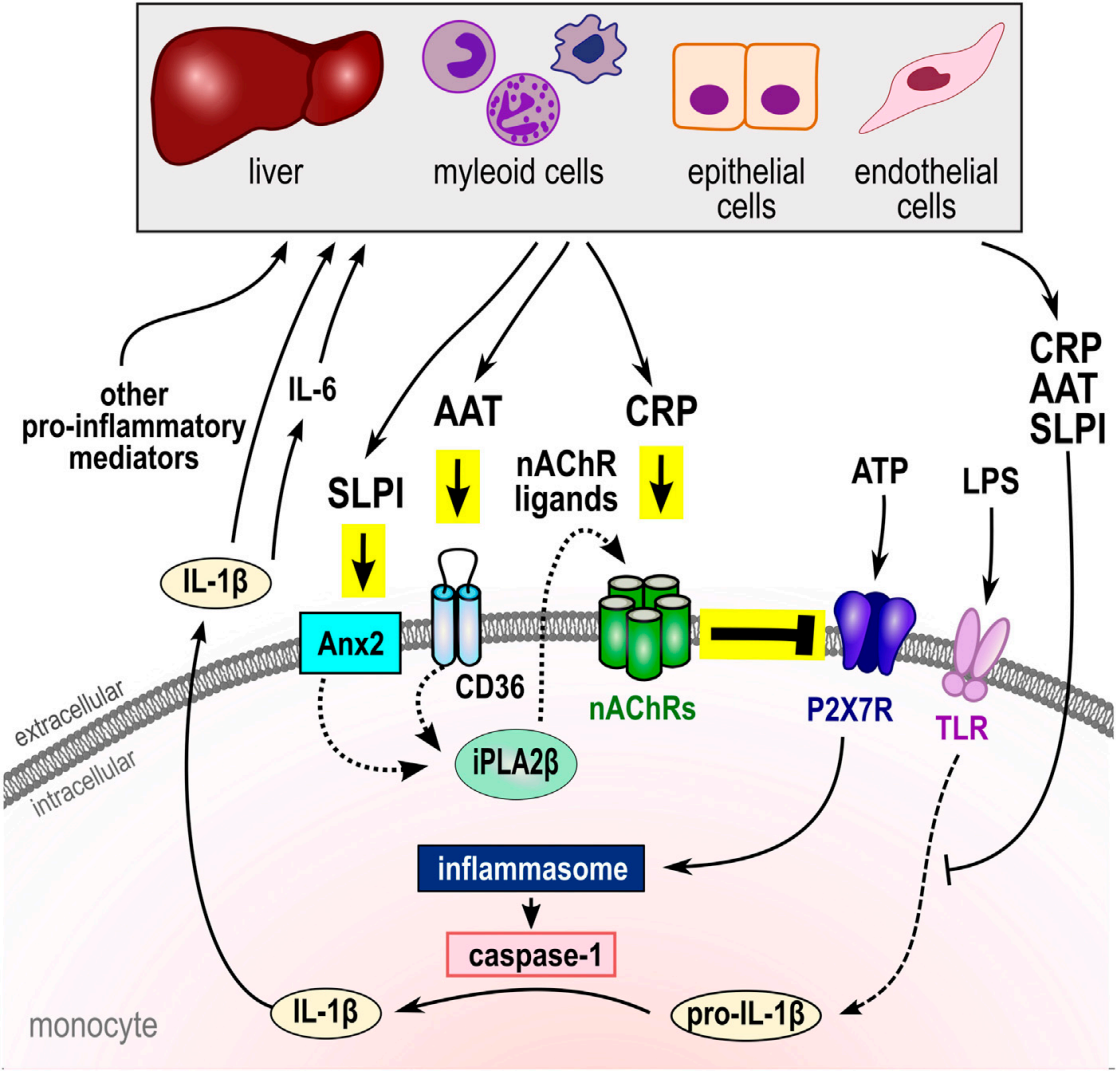
Negative regulation of ATP-induced NLRP3 inflammasome activation and cytokine secretion by the acute-phase proteins CRP, AAT, and SLPI. In monocytes, activation of the P2X7R by extracellular ATP results in the assembly of the NLRP3 inflammasome and activation of capspase-1 that cleaves pro-IL-1β and enables its swift release. IL-1β in turn induces IL-6 and both, like other pro-inflammatory mediators, activate the synthesis of the acute phase proteins CRP, AAT and SLPI by hepatic, myeloid, epithelial, and endothelial cells. There is growing evidence, that CRP, AAT, and SLPI can control the biosynthesis, maturation, and secretion of pro-inflammatory cytokines by controlling cytokine expression levels. Moreover, these acute-phase proteins can control the ATP-induced inflammasome activation and IL-1β release by activation of unconventional monocytic nAChRs containing subunits α7, α9 and/or α10 that inhibit the ionotropic function of the P2X7R. Different nAChR subunits interact, depending on the respective nicotinic agonist (see Table 1). While CRP directly activates nAChRs, AAT (via CD36) and SLPI (via Anx2) activate iPLA2β and induce the release of ligand(s) of nAChRs with yet unknown structure. In summary, apart from other functions, CRP, AAT, and SLPI seem to be central elements of systemic negative feedback loops that protect the host against systemic hyperinflammation, barrier dysfunction, and death by multiple organ damage. AAT, α1-antitrypsin; Anx2, annexin 2 (ANXA2); CRP, native pentameric C-reactive protein; IL, interleukin; LPS, lipopolysaccharide; nAChRs, nicotinic acetylcholine receptor (CHRNA); NLRP3, NACHT, LRR, and PYD domains-containing protein 3; SLPI, secretory leukocyte protease inhibitor; P2X7R, ATP receptor P2X7; iPLA2β, calcium-independent phospholipase A2β (PLA2G6); TLR, Toll-like receptors.

An increasing body of evidence suggests that, in addition to functions in host defense against infections, the acute-phase proteins CRP, AAT, and SLPI are part of anti-inflammatory negative feed-back loops. The anti-inflammatory functions of these acute-phase reactants are pleiotropic and control the biosynthesis of pro-inflammatory cytokines. Furthermore, CRP, AAT, and SLPI inhibit the ATP-induced NLRP3 inflammasome-dependent maturation and release of IL-1β in an at least partially redundant, nAChR- and P2X7R-dependent way (Figure 1). Physiological concentrations of AAT in the blood and SLPI at mucosal surfaces are sufficient to down-regulate IL-1β release in healthy and diseased persons, unless they are counter-regulated by other mechanisms such as those induced by amyloid-β. In contrast, increased CRP blood levels typical for patients with moderate systemic inflammation are needed. Interestingly, CRP, AAT, and SLPI specifically inhibit the ATP (trauma)-induced release of IL-1β, while sparing the ATP-independent inflammasome activation against pathogens, which is vital in the context of severe injuries and infections. More research is needed to fully elucidate the molecular mechanisms involved and to identify the nAChR agonists associated with CRP as well as those released in response to AAT or SLPI, because this may pave the path towards novel anti-inflammatory therapies. If further acute-phase reactants can also activate monocytic nAChRs to control ATP-mediated IL-1β release, remains to be investigated. The observed pleiotropy and redundancy of the acute-phase protein-mediated anti-inflammatory feed-back loops may indicate a strong pressure on the evolution of efficient methods controlling and terminating inflammation caused by traumatic injury. This makes perfect sense, because animals including hominids weakened by ongoing inflammation and corresponding increased levels of acute-phase proteins are typical victims of predators in wildlife. Mechanisms that prevent hyperinflammation in response to a second hit, would offer a considerable survival advantage to those, who escape the attack of a predator but suffer from serious injuries. Clinically, increased levels of acute-phase proteins should not only be regarded as a warning sign but might for instance protect patients from over-shooting systemic inflammation in response to trauma surgery.
